# cAMP-Signalling Regulates Gametocyte-Infected Erythrocyte Deformability Required for Malaria Parasite Transmission

**DOI:** 10.1371/journal.ppat.1004815

**Published:** 2015-05-07

**Authors:** Ghania Ramdani, Bernina Naissant, Eloise Thompson, Florence Breil, Audrey Lorthiois, Florian Dupuy, Ross Cummings, Yoann Duffier, Yolanda Corbett, Odile Mercereau-Puijalon, Kenneth Vernick, Donatella Taramelli, David A. Baker, Gordon Langsley, Catherine Lavazec

**Affiliations:** 1 Laboratoire de Biologie Cellulaire Comparative des Apicomplexes, Faculté de Médicine, Université Paris Descartes—Sorbonne Paris Cité, Paris, France; 2 Inserm U1016, CNRS UMR8104, Institut Cochin, Paris, France; 3 Laboratoire de Biologie de la Transmission de *Plasmodium*, Faculté de Médicine, Université Paris Descartes—Sorbonne Paris Cité, Paris, France; 4 Faculty of Infectious and Tropical Diseases, London School of Hygiene & Tropical Medicine, London, United Kingdom; 5 Institut Pasteur, Unité de Génétique et Génomique des Insectes Vecteurs, CNRS URA 3012, Paris, France; 6 Dipartimento di Scienze Farmacologiche e Biomolecolari (DiSFeB), Università di Milano, Milano, Italy; 7 Institut Pasteur, Unité d’Immunologie Moléculaire des Parasites, CNRS URA 2581, Paris, France; University of Geneva, SWITZERLAND

## Abstract

Blocking *Plasmodium falciparum* transmission to mosquitoes has been designated a strategic objective in the global agenda of malaria elimination. Transmission is ensured by gametocyte-infected erythrocytes (GIE) that sequester in the bone marrow and at maturation are released into peripheral blood from where they are taken up during a mosquito blood meal. Release into the blood circulation is accompanied by an increase in GIE deformability that allows them to pass through the spleen. Here, we used a microsphere matrix to mimic splenic filtration and investigated the role of cAMP-signalling in regulating GIE deformability. We demonstrated that mature GIE deformability is dependent on reduced cAMP-signalling and on increased phosphodiesterase expression in stage V gametocytes, and that parasite cAMP-dependent kinase activity contributes to the stiffness of immature gametocytes. Importantly, pharmacological agents that raise cAMP levels in transmissible stage V gametocytes render them less deformable and hence less likely to circulate through the spleen. Therefore, phosphodiesterase inhibitors that raise cAMP levels in *P*. *falciparum* infected erythrocytes, such as sildenafil, represent new candidate drugs to block transmission of malaria parasites.

## Author Summary

Malaria transmission is ensured by deformable mature gametocyte-infected erythrocytes being taken up when a mosquito bites. Non-deformable immature gametocyte stages are sequestered in the bone marrow, as their lack of deformability would lead to their splenic clearance. In the present study, we apply nano-filtration technology to mimic splenic retention and demonstrate that deformability of transmissible mature stage V gametocytes is regulated by parasite cyclic AMP-dependent kinase signalling. Importantly, when we used drugs to raise cAMP levels we render transmissible mature gametocytes as stiff as non-transmissible gametocytes. In contrast, when we inhibit the cAMP-dependent kinase we render immature gametocytes more deformable. Thus, by two different approaches we confirm that the drop in cAMP levels in mature gametocytes leads to an increase in their deformability and hence more likely to circulate through the spleen. Our molecular observations have the potential to be translated into therapies for blocking malaria transmission by demonstrating that raising cAMP levels with sildenafil also known as “Viagra” renders mature gametocytes rigid. These findings provide the proof of principle that deformability of circulating gametocytes is targetable by pharmacological agents and as such, it provides a novel approach to prevent the spread of parasites. PDE inhibitors therefore represent novel drug leads potentially capable of blocking transmission and improving the worldwide fight to eliminate malaria from the human population.

## Introduction

Recent renewed emphasis on the eradication of malaria has highlighted the need for novel interventions to target the parasite during transmission from the human host to the mosquito. Drug treatments to clear asexual blood stage parasites (that cause pathology) do not kill mature gametocytes and therefore allow transmission to continue [1]. Transmission of malaria parasites relies on the sexual stages, termed gametocytes that circulate in the peripheral blood and are taken up by *Anopheles* mosquitos during a blood meal. For *Plasmodium falciparum*, the causative agent of the most severe form of human malaria, gametocyte maturation requires about 10 days and is divided in five morphological stages [2]. During this period, immature gametocyte-infected erythrocytes (GIE) sequester in internal organs such as bone marrow and spleen [3–6]. Sequestration mechanisms of GIE are still unknown, although failure of immature GIE to adhere to endothelial cell lines *in vitro* [7], and absence on their surface of parasite structures allowing cytoadhesion of asexual stages [8], suggest that GIE-host interactions are unlikely to be mediated by cytoadhesion. Recent evidence rather suggests that GIE biomechanical properties may play an important role in this process [9]. At maturation GIE are released into the blood circulation, where they can persist for several days [10], thus increasing the likelihood of parasites being taken up during a mosquito blood meal and ensuring transmission. This remarkable ability of mature GIE to circulate through the spleen is due to the important deformability that they acquire during the transition between stages IV to V [9,11,12]. By contrast, immature GIE are particularly stiff, which likely contributes to their sequestration by mechanical retention [9]. Therefore, modulation of GIE mechanical properties plays a key role in their microcirculatory behaviour and it has been proposed that interfering with mature GIE filterability through spleen capillaries may represent a novel way to block parasite transmission [4,9]. However, mechanisms mediating the switch in GIE deformability late in the maturation process are still elusive. The disassembly of the microtubule subpellicular network subtending the trilaminar membrane structure in the transition from stage IV to stage V gametocytes probably contributes to this process [12–15]. The switch in deformability is also linked to the de-association of the parasite-derived STEVOR proteins from the infected erythrocyte membrane [9]. These processes must be tightly controlled and signalling likely plays a regulatory role. In uninfected erythrocytes, changes in phosphorylation status, including phosphorylation by cAMP-dependent kinase A (PKA), are known to regulate mechanical properties of the erythrocyte membrane [16,17]. For instance, phosphorylation of band 4.1 by PKA may be central to the regulation of erythrocyte cytoskeletal organization and membrane mechanical properties [18]. PKA phosphorylation of dematin has also been shown to modulate the association between actin and spectrin in the erythrocyte cytoskeleton [19,20]. In infected erythrocytes, PKA activity results from both the human and the parasite enzymes [21]. During the parasite’s life cycle, plasmodial PKA activity is implicated in a wide variety of processes including *P*. *berghei* sporozoite motility and liver cell invasion [22], *P*. *falciparum* erythrocyte invasion by merozoites [23,24], or modulation of infected erythrocyte membrane permeability [25]. So far, there is no evidence for a regulatory role for cAMP-signalling during sexual development. However, adenylate cyclase alpha (*PfACα*) is highly expressed in gametocytes [26], and PKA activity is reportedly higher in gametocyte-producing parasites compared to parasites defective in gametocyte production [27], suggesting a potential role for cAMP-signalling in sexual development.

Here, we have investigated the role of cAMP-signalling in modulating GIE mechanical properties. Using both genetic and pharmacological manipulation of cAMP signalling in conjunction with the microsphiltration method to assess the ability of GIE to circulate through inter-endothelial splenic slits, we show that a decrease in cAMP levels increases mature GIE deformability, and conversely, increasing cAMP levels increases GIE stiffness. These findings provide the proof of principle that molecules targeting phosphodiesterases (PDE) represent a novel drug class capable of blocking malaria transmission.

## Results

### PfPKA-mediated phosphorylation contributes to immature GIE stiffness

**Fig 1:**
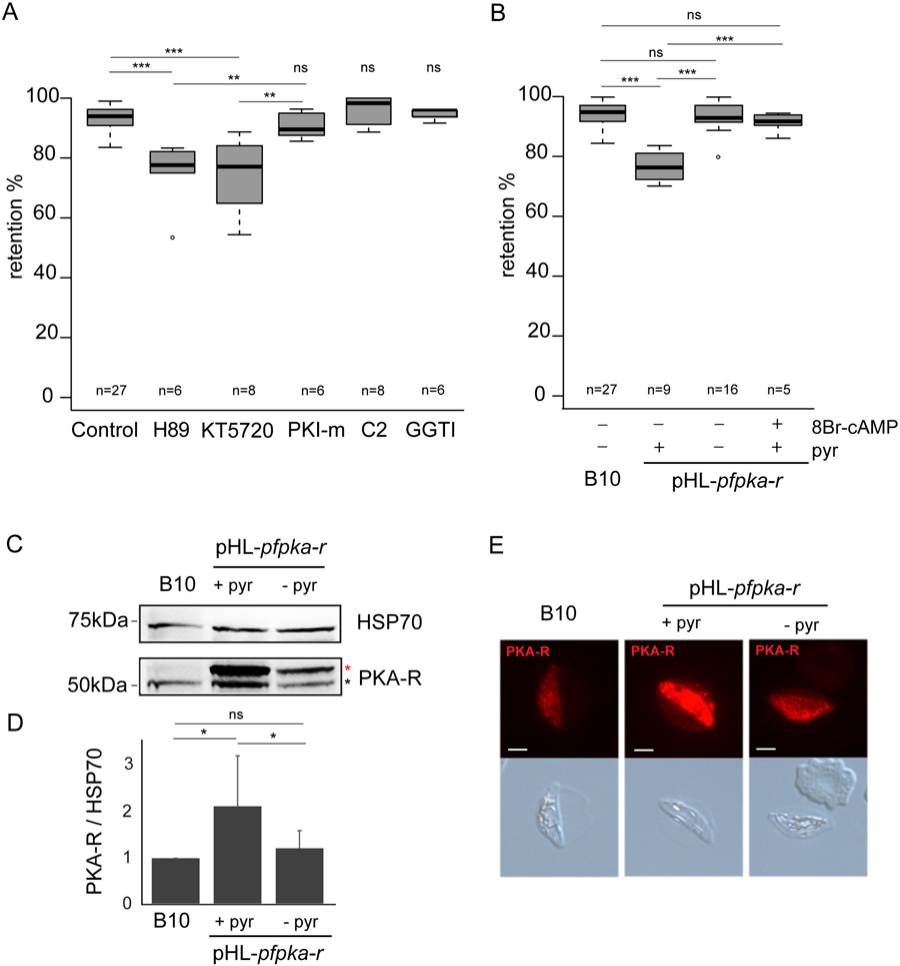
*Pf*PKA-mediated phosphorylation contributes to immature GIE stiffness. **A.** Retention in microsphilters of stage III GIEs from the B10 clone pre-incubated 30 min to 1 h at 37°C with 10 μM H89, 10 μM KT5720, 10 μM PKI-m, 10 μM compound 2, 10 μM GGTI-298 or 0,1% DMSO (Control). Error bars denote the standard error of the mean. *** and ** Highly significant differences in retention rates (*** *P* < 0.001; **P < 0.01); ns: non-significant differences in retention rates compared to control; n: number of experiments. Outliers are shown as open circles. **B.** Retention in microsphilters of stages III GIEs from the B10 clone and the pHL-*pfpka-r* clone cultivated with and without pyrimethamine for 15 generations. The pHL-*pfpka-r* clone was pre-incubated 15 min at 37°C with 100μM 8Br-cAMP. Error bars denote the standard error of the mean. ***Highly significant differences in retention rates (*P <* 0.001); ns: non-significant differences in retention rates; n: number of experiments. Outliers are shown as open circles. **C**. Western-blot analysis of *Pf*PKA-R expression in stage III GIE from the B10 clone and the pHL-*pfpka-r* clone cultivated in presence (+ pyr) or absence (- pyr) of pyrimethamine. Immunoblots were probed with rabbit polyclonal antibodies directed against *Pf*PKA-R and with a mAb directed against *Pf*HSP70 to normalize expression. Black star indicates the expected size for *Pf*PKA-R (50.8 kDa); Red star indicates *Pf*PKA-R with post-translational modifications. The experiment has been performed seven times. Error bars denote the standard error of the mean. *Significant differences in phosphorylation signal (*P < 0.05); ns: non-significant differences in phosphorylation signal. **D**. Quantitation of signal intensities in panel C using Quantity One software (BioRad). Analysis shows a 1.6-fold increase in *Pf*PKA-R expression in the pHL-*pfpka-r* clone (+ pyr) compared to B10. Decrease of *Pf*PKA-R expression in absence of pyrimethamine (- pyr) indicates a loss of episomal expression of the *Pf*PKA-R protein. **E.** Immunofluorescence analysis of stage III GIE from the B10 clone and the pHL-*pfpka-r* clone cultured for 15 generations in the presence (+ pyr) or absence (- pyr) of pyrimethamine. Infected erythrocytes were stained with anti-*Pf*PKA-R antibodies followed by anti-rabbit Alexa 594-conjugated IgG. Pictures were taken under identical exposure conditions. The bars represent 2 μm.

To investigate whether PKA activity modulates GIE mechanical properties, we assessed the filterability of GIE using the microsphiltration method, which mimics the physical constraints experienced by infected erythrocytes in the splenic microcirculation [28,29]. In this system, increased retention rates correspond to decreased erythrocyte deformability and impaired filterability. We treated stage III GIE with KT5720 and H89, two independent and widely used PKA inhibitors that also block a few other kinases [30,31] and that have already been shown to inhibit PKA activity in *P*. *falciparum* [21,24,32]. Before incubation with these inhibitors, approximately 94% of stage III GIE and 30% of stage V GIE were retained on the microspheres (Fig 1A and S1 Fig), confirming the retention rates previously observed [9]. Importantly, incubation with H89 and KT5720 significantly decreased the retention rates of stage III GIE (*P* = 3.10e-6 and 6.10e-6 for H89 and KT5720, respectively; Fig 1A) consistent with PKA activity contributing to immature GIE stiffness, whereas incubation of stage V GIE with H89 did not alter their retention rates (S1 Fig). By contrast, stage III GIE retention rates were not affected upon incubation with compound 2, a highly selective inhibitor of apicomplexan cGMP-dependent protein kinase (PKG) [33], or with GGTI-298, an inhibitor of the cAMP-effector exchange protein activated by cAMP (EPAC) [34]. Furthermore, GIE filterability was not affected upon incubation with PKI-m, a membrane permeable inhibitor of the human PKA (PKA) that is a poor inhibitor of parasite PKA (*Pf*PKA) [32]. This suggests that immature GIE filterability is modulated by *Pf*PKA, and not by human erythrocyte PKA.

To confirm this notion, we measured retention rates of a transgenic parasite that exhibits a down-regulation in *Pf*PKA activity due to episomal overexpression of the regulatory (*Pf*PKA-R) subunit of *Pf*PKA (pHL*-pfpka-r*) [25]. cAMP binding to PKA-R liberates the catalytic subunit (PKA-C) from inactive R/C complexes and over-expression of PKA-R acts as a cAMP sink dampening complex dissociation, so decreasing *Pf*PKA activity. Immunoblotting and immunostaining of gametocytes with specific antibodies indicated that *Pf*PKA-R expression was significantly increased in pHL*-pfpka-r* transgenic parasites (Fig 1C, 1D and 1E). We note that two PfPKA-R specific bands are identified in gametocytes compared to a single band in schizonts [35], indicating that the R subunit undergoes translational modification during gametocytogenesis. Microsphiltration analysis of pHL*-pfpka-r* immature GIE showed a significant decrease in retention rates, similar to that observed upon incubation of wild-type GIE with H89 (*P* = 0, Fig 1B). To confirm that decreased retention rates were due to dampened cAMP-signalling, we measured deformability of pHL*-pfpka-r* immature GIE following incubation with the cell permeable, phosphodiesterase resistant cAMP analogue, 8Br-cAMP; raising cAMP levels restored retention rates to wild-type phenotype (Fig 1B).

Loss of episomally derived *Pf*PKA-R over-expression in pHL*-pfpka-r* transgenic parasites should result in a regain in *Pf*PKA activity and restoration in the levels of retention associated with endogenous *Pf*PKA expression. To promote shedding of the episome-encoded R subunit, the pHL*-pfpka-r* transgenic line was cultured for several generations without pyrimethamine selection required to retain the episome, leading to a new line called pHL*-pfpka-r-wt*. Immunoblotting and immunostaining of stage III GIE with specific anti-*Pf*PKA-R antibodies confirmed that *Pf*PKA-R expression in pHL*-pfpka-r-wt* had reverted to that of wild type levels, consistent with loss of the episome harboring the *pfpka-r* expression cassette (Fig 1C, 1D and 1E). Retention rates of pHL*-pfpka-r*-*wt* immature GIE were similar to wild-type GIE, indicating that the retention phenotype mediated by over-expressed *Pf*R-induced down-regulation of *Pf*PKA activity had reverted (Fig 1B). These results indicate that *Pf*PKA-mediated phosphorylation contributes to immature GIE stiffness.

### Phosphorylation events contribute to the switch in deformability

**Fig 2:**
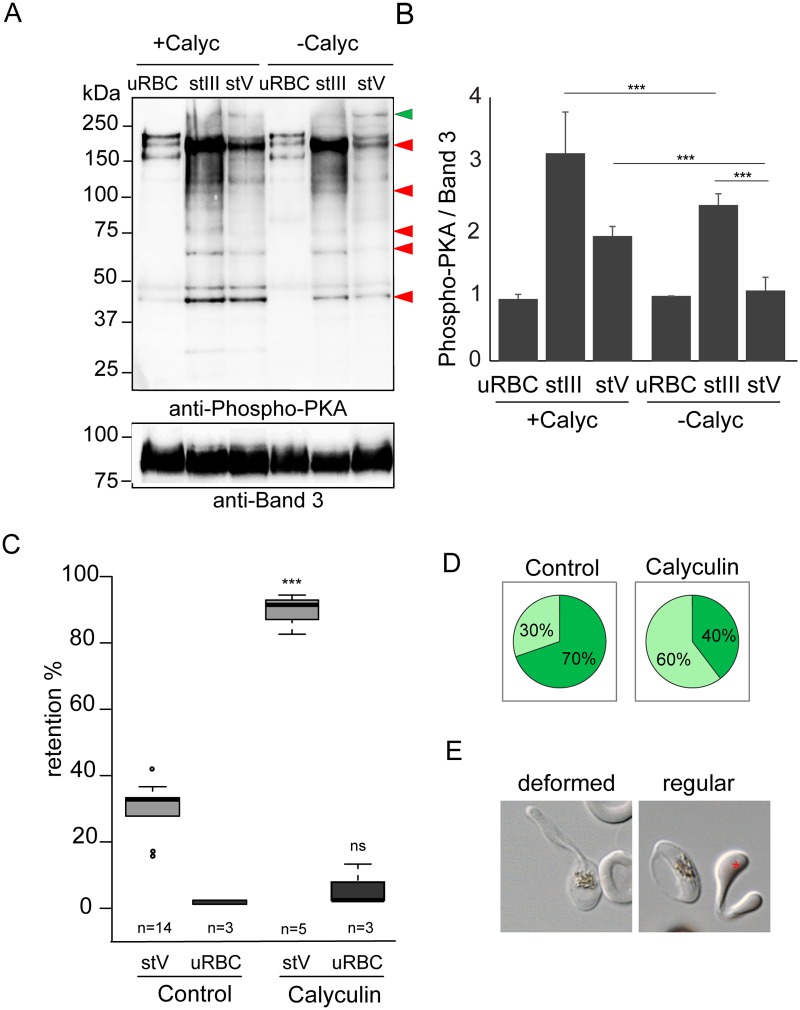
Phosphorylation events contribute to the switch in deformability. **A.** Western-blot analysis of Phospho-PKA site (RRXS*/T*) expression in uninfected red blood cells (uRBCs), MACS-purified stage III (stIII) and stage V (stV) GIE from the B10 clone treated (+Calyc) or not (-Calyc) with 50 nM calyculin during 2h at 37°C. Analysis was performed on membrane extracts recovered by centrifugation after 1% Triton X100 treatment. Immunoblot was probed with rabbit monoclonal antibody directed against phospho-PKA sites (RRX*S/*T) and with mouse mAb directed against Band 3 to normalize expression. Red arrows show bands with more intense phosphorylation signal in stage III GIE than in stage V GIE. Green arrow show band with less intense phosphorylation signal in stage III GIE than in stage V GIE. The experiment has been performed three times. Error bars denote the standard error of the mean. ***Highly significant differences in phosphorylation signal (*P <* 0.001). **B.** Quantitation of signal intensities in panel D using Quantity One software (BioRad). The strength of the phosphorylation signal in untreated uninfected red blood cells lysate was set to 1 and all other signals are relative to that. **C.** Retention in microsphilters of stages V GIEs (light grey), or uninfected red blood cells (uRBCs, dark grey). GIEs were pre-incubated at 37°C for 2 h with 50 nM calyculin, or 0.5% DMSO (control). ***Highly significant differences in retention rates compared to control (*P <* 0.001); ns: non-significant differences in retention rates compared to control; n: number of experiments. **D**. Graphical representation for the proportion of GIE showing a regular (light green), or deformed (dark green) shape in a population of paraformaldehyde-fixed GIE, as they flow through the microsphilters after pre-incubation at 37°C 2 h with 50 nM calyculin, or 0.5% DMSO (control). **E.** Differential interference contrast images of paraformaldehyde-fixed GIE, as they flow through the microsphilters. A majority of DMSO-treated GIE (left panel) are twisted and deformed, whereas inhibitor-treated GIE (right panel) keep a regular shape, unlike uninfected erythrocytes (red star).

To further demonstrate the contribution of *Pf*PKA to immature GIE stiffness we probed membrane extracts of stage III and stage V GIE using a monoclonal antibody specific for canonical phospho-PKA sites (RRXS*/T*) (Fig 2A and 2B). The intensity of phosphorylation was 2-fold lower in stage V GIE compared to stage III, indicating that membrane components were less phosphorylated by PKA in mature than in immature gametocytes. At least five proteins displayed a higher degree of PKA site phosphorylation in stage III compared to stage V, suggesting that these PKA substrates are potentially involved in mediating the membrane rigidity phenotype (Fig 2A and 2B). Reduced PKA phosphorylation at stage V indicates a drop in cAMP levels accompanies the switch in deformability that occurs at the transition between immature and mature stages. The degree of cAMP-mediated phosphorylation can be increased by treatment with calyculin A, a serine/threonine phosphatase inhibitor (known to inhibit *P*. *falciparum* phosphatase-1-like activities [36,37]) that diminishes dephosphorylation of PKA substrates. Incubation of stage V GIE with calyculin A increased phosphorylation intensity at 2-fold to levels observed in stage III (Fig 2A and 2B). Consistently, incubation of stage V GIE with calyculin A markedly impaired their filterability by increasing the retention rates in microspheres up to 90% (*P* = 0.0038), whereas it did not significantly affect filterability of uninfected erythrocytes (*P* = 0.7; Fig 2C). To validate that increased retention rates triggered upon incubation with calyculin A corresponded to a decrease in GIE deformability, we visualized the shape of GIE as they flowed through the matrix by adding a paraformaldehyde-fixation step to the microsphiltration experiment (Fig 2D and 2E). 30% of untreated GIE maintained their original shape, whereas 70% displayed a twisted and deformed shape, likely reflecting their ability to squeeze and slide between microspheres [9]. Upon incubation with calyculin A the proportion of deformed GIE decreased from 70% to 40% (Fig 2D and 2E). To rule out any cytotoxic effect of calyculin A on gametocytes that could un-specifically result in higher cell rigidity, we measured parasite lactate dehydrogenase activity immediately and 72 h after calyculin A treatment and validated that gametocyte viability was not affected (Table 1). Manipulating the phosphorylation status using calyculin A clearly affects GIE mechanical properties and given the documented effect of calyculin A on PKA activity in other systems [37], it is consistent with *Pf*PKA activity in mediating gametocyte deformability.

**Table 1:** Viability of 3D7 stage V GIE treated with different inhibitors.

Treatment	Dose	PreTreatment min	pLDH (time 0) % Controls ±SD	pLDH (after 72 h) % Controls±SD
Medium			100	100
Sildenafil citrate	100 μM	30	117.95 ± 5.4	99.07 ± 7.5
H89	10 μM	30	94.44 ± 11.8	86.41 ± 7.1
GGTI298	10 μM	30	105.81 ± 12.1	94.89 ± 4.6
Zaprinast	65 μM	30	112.16 ± 15.7	83.72 ± 7.9
Calyculin A	50 nM	120	124.01 ± 10.5	84.91 ± 8.7
8Br-cGMP	150 μM	30	103.20 ± 28.8	86.97 ± 10.6
Forskolin	150 μM	30	94.83 ± 14.6	95.92 ± 25.1

3D7 Stage V GIE were treated with the inhibitors for the indicated times, then washed and their viability evaluated with the colorimetric pLDH assay either immediately (time 0) or after 72 h incubation at 37°C, as described [56]. The data are expressed as percent of untreated controls and are the mean of two experiments in quadruplicate.

### GIE filterability is dependent on cAMP concentration

**Fig 3:**
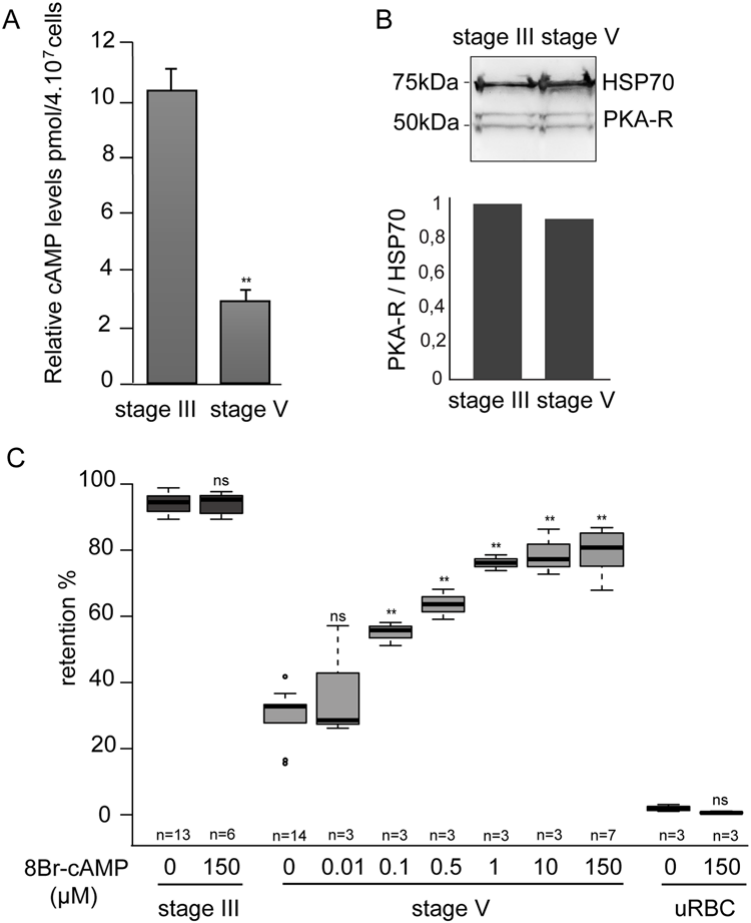
GIE filterability is dependent on cAMP concentration. **A.** cAMP concentration drops in mature GIE. The total intracellular cAMP concentration was measured in stage III and V GIE using a competitive immunoassay for the quantitative determination of cAMP. GIE were harvested by magnetic isolation and aliquots of 6.10^6^ cells were assayed in duplicate wells. The assay was carried out three times. Error bars denote the standard error of the mean. **Highly significant difference compared to stage III GIE (*P <* 0.01). **B**. Western-blot analysis of PKA-R expression in MACS-purified stage III and stage V GIE (5.10e6 parasites/lane). Immunoblots were probed with rabbit polyclonal antibodies directed against *Pf*PKA-R and with a mAb directed against *Pf*HSP70 to normalize expression. Quantity One (BioRad) analysis shows that *Pf*PKA-R levels were not significantly different between stage III and stage V. **C**. Retention rates in microsphilters of stage III GIE (dark grey), stage V GIE (light grey) and uninfected red blood cells (uRBC, black) pre-incubated 15 min at 37°C with different concentrations of 8Br-cAMP. Error bars denote the standard error of the mean. Outliers are shown as open circles. **Highly significant differences in retention rates compared to control without 8Br-cAMP (*P <* 0.01); ns: non-significant differences in retention rates compared to control; n: number of experiments.

The above results suggest that changes in cellular cAMP levels influence GIE deformability via PKA activation. We first measured cAMP concentrations in MACS-purified GIE at immature and mature stages. Intracellular levels of cAMP decrease approximately five-fold in stage V GIE compared to stage III GIE (*P* = 0.008; Fig 3A), while *Pf*PKA-R levels are unaltered between immature and mature stages (Fig 3B). Thus, increased deformability of stage V GIE is accompanied by reduced PKA activity due to a decrease in cAMP levels. This notion was underscored by increasing cAMP levels via addition of 8Br-cAMP and measuring the filterability of stage III compared to stage V gametocytes. Upon incubation with 8Br-cAMP, retention rates of stage III GIE were not modified, whereas those of stage V GIE were proportionally augmented with increasing concentrations of 8Br-cAMP. High concentrations of 8Br-cAMP did not affect filterability of uninfected erythrocytes, indicating that 8Br-cAMP specifically affects *P*. *falciparum* mature GIE (Fig 3C). Analysis of Giemsa stained smears of stage V GIE upstream and downstream of the microsphere matrix showed unaltered male:female ratios indicating that both male and female gametocytes use cAMP to regulate infected cell deformability.

### cAMP degradation by phosphodiesterases regulates GIE mechanical properties

**Fig 4:**
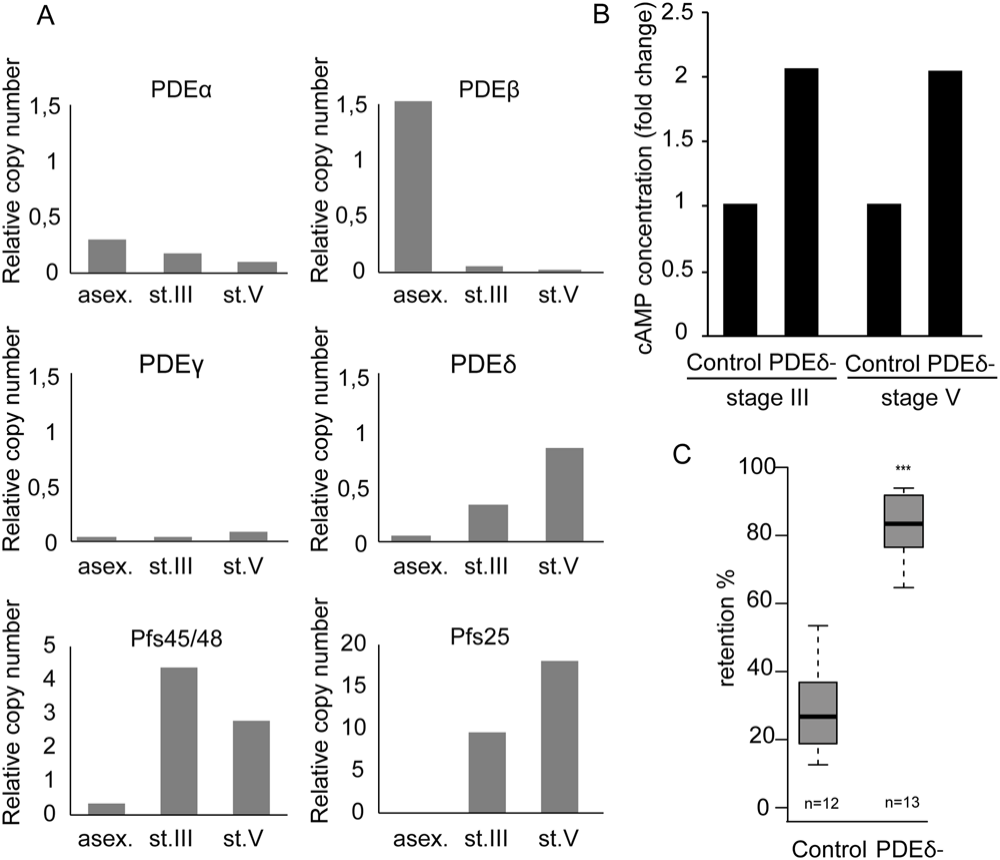
cAMP degradation by phosphodiesterases regulates GIE mechanical properties. **A.** mRNA levels of four *PDE*s were determined by real time RT-PCR in asexual blood-stages (asex), stage III GIE (st.III) and stage V GIE (st.V). Relative amounts of transcript were normalized to mRNA levels of the *PfHK* (*PF08_0085)*, *Pfs48/45* (*PF13_0247)* and *Pfs25* (*PF10_0303)* were used as markers of stage III and stage V GIE, respectively. Triplicate PCR reactions were analysed for each sample. **B.** The total intracellular cAMP concentration was measured in stage III and V GIE from the 3D7 clone and the *PfPDEδ-*mutant clone 4 using a competitive immunoassay for the quantitative determination of cAMP. GIE were harvested by magnetic isolation and aliquots of 6.10^6^ cells were assayed in triplicate wells. The assay was carried out at least three times for each clone. **C.** Retention in microsphilters of stages V GIEs from the 3D7 clone and the *PfPDEδ-*mutant clone 4. Error bars denote the standard error of the mean. Outliers are shown as open circles. n: number of experiments.

The drop in cAMP concentration in stage V GIE might be a result of either reduced cAMP synthesis by adenylate cyclases, or increased degradation by phosphodiesterases (PDEs). In *P*. *falciparum*, four genes encode PDEs: *PfPDEα* (PF3D7_1209500.1), *PfPDEβ* (PF3D7_1321500.1), *PfPDEγ* (PF3D7_1321600) and *PfPDEδ* (PF3D7_1470500) [38,39]. We performed real-time RT-PCR to quantify mRNA levels for all *PDE*s in asexual blood-stages as well as in immature and mature gametocytes. *PfPDEα* and *PfPDEβ* are mainly expressed in asexual blood-stages, *PfPDEγ* is minimally expressed in all blood-stages, whereas *PfPDEδ* is highly expressed in stage V gametocytes (Fig 4A). Importantly, mRNA levels of *PfPDEδ* increase approximately two- to three-fold in stage V compared to stage III gametocytes. These results are consistent with expression data available at PlasmoDB (http://www.plasmodb.org/), where *PfPDEδ* is annotated as almost exclusively expressed in stage V gametocytes [40,41].

To determine whether *Pf*PDEδ is involved in triggering the switch in deformability observed in stage V GIE, we analysed retention rates for mature GIE from transgenic parasites in which the *PfPDEδ* gene had been deleted [42]. We first measured cAMP concentration in MACS-purified stage III and stage V GIE and found loss of PfPDEδ activity led to a two-fold increase in cAMP levels in both immature and mature GIE (Fig 4B). Microsphiltration analysis of mature GIE from the *PfPDEδ-*mutant line showed a significant increase in retention rates (Fig 4C), indicating that this phosphodiesterase participates in regulating cAMP levels in mature gametocytes and hence GIE deformability.

### Pharmacological perturbation of cellular cAMP levels impairs mature GIE filterability

**Fig 5:**
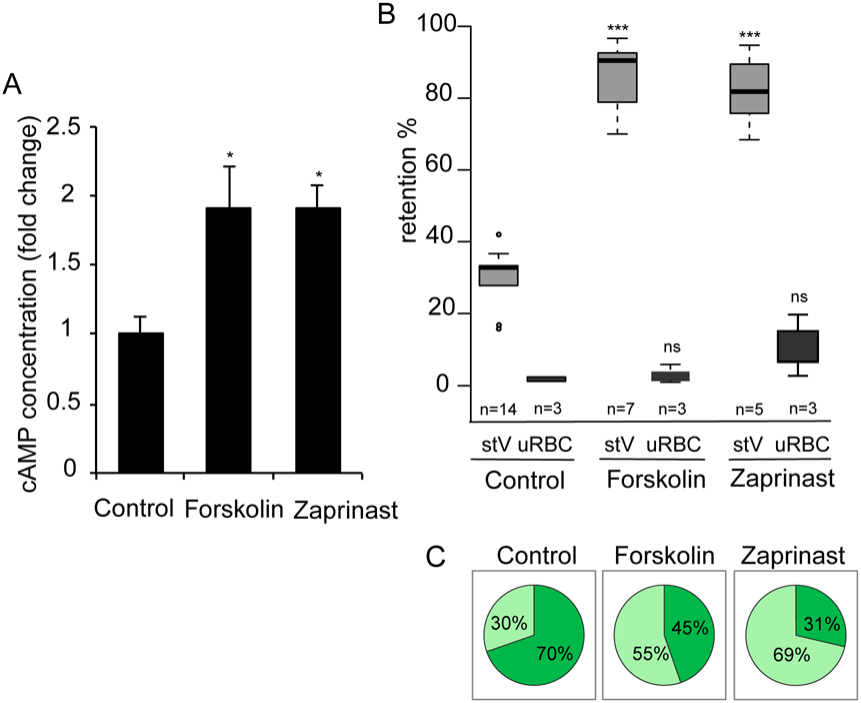
Interfering with cAMP levels impairs mature GIE filterability. **A.** Stage V GIE were harvested by magnetic isolation and incubated at 37°C for 30 min with 150 μM forskolin, 65 μM zaprinast, or 0.1% DMSO (Control). The total intracellular cAMP concentration was measured on aliquots of 6.10^6^ cells in duplicate wells. The assay was carried out three times. Error bars denote the standard error of the mean. *Significant differences in retention rates compared to control (*P <* 0.05). **B.** Retention in microsphilters of stages V GIEs (light grey), or uninfected red blood cells (uRBCs, dark grey). GIEs were pre-incubated at 37°C for 30 min with 150 μM forskolin, 65 μM zaprinast or 0,1% DMSO (control). ***Highly significant differences in retention rates compared to control (DMSO) (*P <* 0.001); ns: non-significant differences in retention rates compared to control. n: number of experiments. **C.** Graphical representation for the proportion of GIE showing a regular (light green), or deformed (dark green) shape in a population of paraformaldehyde-fixed GIE, as they flow through the microsphilters after pre-incubation at 37°C for 30 min with 150 μM forskolin, 65 μM zaprinast or 0.1% DMSO (control).

As an alternative way to investigate the effects of raising cAMP levels in GIE, we used pharmacological agents such as forskolin, an activator of mammalian adenylate cyclase, and zaprinast, an inhibitor of PDEs. Although zaprinast is well known as an inhibitor of mammalian PDE5, a cGMP phosphodiesterase, it is known to increase cAMP levels in human erythrocytes [43], and can inhibit both cAMP- and cGMP-PDE activities in *P*. *falciparum* (Table 2) [39]. To validate the effect of these molecules on intracellular levels of cAMP, we measured its concentration in MACS-purified stage V GIE and found a two-fold increase in cAMP levels in cells treated with either compound (*P* = 0.029 and 0.024 for forskolin and zaprinast, respectively; Fig 5A). Importantly, incubation of stage V GIE with either forskolin or zaprinast markedly increased microsphere retention rates by up to 82% (*P* = 0) and 86% (*P* = 0.0002), respectively. Both reagents showed no significant effect on filterability of uninfected erythrocytes (*P* = 0.49 and 0.20 for forskolin and zaprinast, respectively; Fig 5B). Upon incubation with forskolin or zaprinast, the proportion of paraformaldehyde-fixed mature GIE that exhibit a deformed shape as they flowed through the matrix was decreased to 45% and 31%, respectively, compared to 70% of untreated cells (Fig 5C). The ensemble indicates that pharmacological agents that raise levels of cAMP affect mature GIE deformability impairing their ability to pass through an *in vitro* model for splenic filtration.

**Table 2:** The effect of PDE inhibitors on particulate fractions isolated from *P*. *falciparum* parasites.

	Target PDE class in humans	Pf cAMP-PDE	Pf cGMP-PDE	Tb PDE1	Bovine PDE	Human PDE
IBMX	Non-selective	> 200	> 200	> 1000	1.89 ± 4.38	3.95*
Rolipram	PDE 4 specific (cAMP-specific)	> 200	> 200	280	> 200	2
Zaprinast	PDE 5 and 6 (cGMP-specific)	12.26 ± 1.23	3.03 ± 1.25	> 100	25.21 ± 1.71	0.5
Sildenafil	PDE 5 specific (cGMP-specific)	51.08 ± 1.59	22.52 ± 1.76	1	6.81 ± 1.29	0.0039

IC_50_ values (the concentration of compound that gave 50% inhibition of native PDE activity) are mean ± SEM in μM and were calculated using the Prism software package (GraphPad Software, Inc.). Percentage inhibition data were fitted to a sigmoidal dose-response curve using non-linear regression. Bovine control PDE column displays cAMP dependent activity. All TbPDE1 and most Human values obtained from Kunz, *et al*.[58].

*Value obtained from Tang, *et al*.[59].

>200, sufficient inhibition was not observed for an IC_50_ calculation.

Tb: *Trypanosama brucei*.

**Fig 6:**
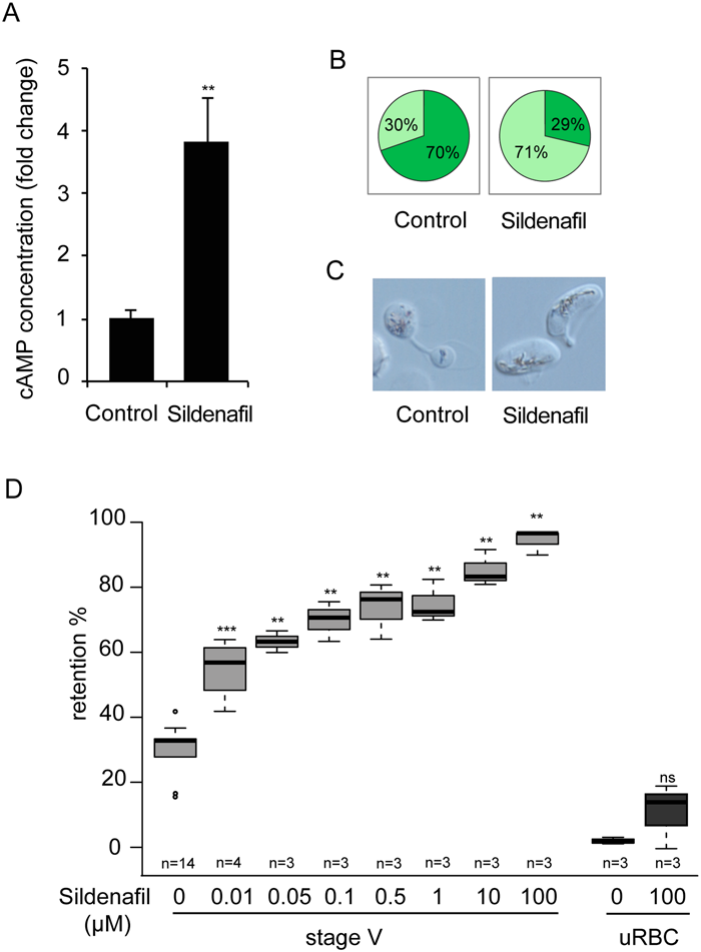
Sildenafil impairs mature GIE filterability. **A**. Stage V GIE were harvested by magnetic isolation and incubated at 37°C for 30 min with 100 μM sildenafil, or 0.1% DMSO (Control). The total intracellular cAMP concentration was measured on aliquots of 6.10^6^ cells in duplicate wells. The assay was carried out five times. Error bars denote the standard error of the mean. **Highly significant differences in retention rates compared to control (DMSO) (*P <* 0.01). **B.** Graphical representation for the proportion of GIE showing a regular (light green), or deformed (dark green) shape in a population of paraformaldehyde-fixed GIE, as they flow through the microsphilters after pre-incubation at 37°C for 30 min with 100μM sildenafil, or 0.1% DMSO (Control). **C**. Differential interference contrast images of paraformaldehyde-fixed GIE, as they flow through the microsphilters. **D**. Retention rates in microsphilters of stage V GIE (light grey) and uninfected red blood cells (uRBC, dark grey) pre-incubated 30 min at 37°C with different concentrations of sildenafil. Error bars denote the standard error of the mean. Outliers are shown as open circles. *** and **Highly significant differences in retention rates compared to control (**P < 0.01; ***P < 0.001); ns: non-significant differences in retention rates compared to control. n: number of experiments.

PDEs have been well studied as potential drug targets in relation to numerous diseases. For example, the PDE5 and PDE6 inhibitor sildenafil (Viagra) is a widely used to treat erectile dysfunction. As a first step to address the potential of sildenafil to impair the circulation of *P*. *falciparum* mature gametocytes in humans and thereby block malaria transmission to mosquitoes, we measured the cAMP concentration in MACS-purified stage V GIE following incubation with sildenafil. We found that 100 μM sildenafil triggered an approximately four-fold increase of intracellular levels of cAMP, leading to levels similar to those of immature GIE (*P* = 0.0068; Fig 6A). An increase in cGMP levels was also measured following sildenafil treatment (S2 Fig), consistent with our observations that both zaprinast and sildenafil can inhibit both cAMP and cGMP hydrolytic activities (Table 1). Upon incubation with different concentrations of sildenafil from 10 nM to 100 μM, retention rates of stage V GIE increased in a dose-dependent manner and reached 92% retention at 100 μM (Fig 6D). At 100 μM the proportion of paraformaldehyde-fixed GIE that exhibit a deformed shape as they flowed through the matrix decreased to 29%, compared to 70% of untreated cells (Fig 6B and 6C). Importantly, mature GIE exhibited greatly reduced filterability with more than 75% retention with 1 μM sildenafil (666.7 ng/ml), which approximately corresponds to the reported peak serum concentration reached in humans after 60 min following 100 mg oral dose (C_max_ 440 ng/ml) [44,45]. The filterability of uninfected erythrocytes was not significantly affected at these sildenafil concentrations indicating that it specifically affects infected erythrocytes (*P* = 0.7012; Fig 6D).

## Discussion

A novel paradigm that recently emerged postulates that the dynamics of immature *P*. *falciparum* GIE sequestration in the extravascular spaces of bone marrow and release of mature forms into peripheral circulation through bone marrow endothelial slits depends on an increase in GIE deformability at the transition from immature to mature stages [6,9]. The ability of mature *P*. *falciparum* GIE to circulate and pass through narrow blood capillaries and splenic slits depends on the deformability of both the parasites and the infected erythrocytes. Here, we have provided new mechanistic insight into the regulation of GIE deformability, and importantly, by demonstrating that GIE deformability is altered by zaprinast and sildenafil treatments we provide the proof of concept that PDE inhibitors could open new avenues towards the design of malaria transmission-blocking drugs.

Stiffer immature gametocyte stages have high levels of cAMP, but become more deformable upon inhibition of *Pf*PKA either pharmacologically or by overexpression of the *Pf*PKA regulatory subunit. Deformability was not affected upon incubation with GGTI-298, making unlikely that cAMP regulates GIE stiffness via activation of *Pf*EPAC [35]. So one mechanism by which raising cAMP levels induce GIE stiffness is via activation of *Pf*PKA and its possible substrates could be parasite proteins involved in mediating the deformability of the parasite itself, a parasite protein(s) that remodels the erythrocyte membrane, or a protein(s) of erythrocyte origin. For instance, glideosome associated protein (GAP) 45 and myosin A, which are components of the microtubule subpellicular network subtending the trilaminar membrane structure in immature gametocytes, have been identified as PKA substrates in asexual stages [12,46]. In uninfected erythrocytes PKA-mediated phosphorylation of dematin and protein 4.1 decreases their ability to promote spectrin binding to F-actin, therefore modulating mechanical properties of the erythrocyte [18,20]. In GIE these proteins may be phosphorylated by *Pf*PKA in immature stages and may become dephosphorylated by parasite phosphatases in deformable stage V gametocytes. The effect on deformability of the phosphatase inhibitor calyculin A is consistent with dephosphorylation playing a role. The detection of five bands showing a decrease in PKA site phosphorylation in mature stages suggests that more than one PKA substrate likely contributes to regulation of GIE deformability. Besides proteins from the erythrocyte cytoskeleton, parasite-encoded STEVOR proteins are also attractive candidates to be *Pf*PKA substrates. They have been shown to impact on the deformability of erythrocytes infected with sexual and asexual *P*. *falciparum* parasites, and the switch in deformability during gametocyte maturation is linked to a de-association of STEVOR proteins from the erythrocyte membrane in mature stages [9,47]. Interestingly, three serine residues and one threonine residue conserved across the entire STEVOR family are predicted to be PKA sites (cbs.dtu.dk/services/NetPhosK). Thus, phosphorylation of STEVOR proteins and its impact on STEVOR subcellular localisation and/or their interaction with erythrocyte cytoskeleton could be dependent on PKA-mediated phosphorylation and future studies will address this point. In addition, the mature parasite-infected erythrocyte surface antigen (MESA) is also expressed in sexual stages and interacts with the erythrocyte cytoskeleton, where it binds to protein 4.1 [48,49]. The presence of four classical PKA phosphorylation sites in MESA suggests that this protein might also be a target of PKA-mediated changes in GIE mechanical properties.

*Pf*PKA-mediated phosphorylation of proteins associated with the erythrocyte cytoskeleton, or parasite proteins located in the erythrocyte membrane implies that the parasite kinase might be exported into the erythrocyte cytosol. *Pf*PKA-R and *PfP*KA-C sequences do not have a recognizable PEXEL/HT motif and whether they are secreted remains hypothetical; however, there are proteins that lack a clear secretion signature that make up the non-PEXEL exportome [50]. Furthermore, in the absence of compelling secretion data for *Pf*PKA-R and *Pf*PKA-C, we entertain the possibility that *Pf*PKA effects on erythrocyte membrane deformability may be mediated indirectly, through *Pf*PKA-dependent phosphorylation of other, as yet to be identified, secreted parasite effectors.

We found that the overall GIE cAMP concentration drops at the transition between immature and mature gametocyte stages, concomitant with the switch in deformability necessary for transmission. We established that raising cAMP levels in stage V GIE with forskolin, zaprinast or sildenafil rendered them stiff, like immature GIE. Zaprinast and sildenafil treatment of stage V GIE clearly led to a rise in cAMP levels and sildenafil also induced a small increase in the amount of cGMP (S2 Fig). Consistent with these observations, retention rates of stage V GIE proportionally augmented with increasing concentrations of 8Br-cGMP (S2 Fig). However, cGMP levels do not change between stage III and V gametocytes [42] and moreover, inhibition of the cGMP-dependent protein kinase (*Pf*PKG) with the specific inhibitor compound 2 did not alter stage III GIE retention rates at a concentration 20-fold higher than required to inhibit erythrocyte invasion by merozoites [33]. Nonetheless, cGMP could impact indirectly on GIE deformability via crosstalk with cAMP signalling, as already reported for uninfected erythrocytes [43]. These observations validate the use of sildenafil to increase mature GIE stiffness.

Several PDE inhibitors have been developed and used as therapeutic agents, and PDE5 has received considerable attention over the last 10 years, with three selective inhibitors now on the market (sildenafil, vardenafil, and tadalafil) [45]. In humans sildenafil acts by inhibiting both PDE5 and PDE6, and we found that in *P*. *falciparum*-infected erythrocytes this inhibitor clearly led to a rise in cAMP levels. Consistently, it increased GIE retention in an *in vitro* model for splenic filtration, demonstrating that administration of PDE inhibitors could be a new way to block parasite transmission to mosquitoes. Interestingly, previous studies with *PfPDEδ-*mutant line pointed to an essential role for cGMP-signalling in *P*. *falciparum* gametogenesis and ookinete formation in the mosquito vector [42,51]. This suggests that inhibition of plasmodial PDEs with sildenafil or derived analogues has the potential to raise both cAMP and cGMP levels resulting in a block in both gametocyte transmission via changes in GIE deformability and on ookinete development in mosquitoes.

Our observations provide an opportunistic approach towards the discovery of new malaria transmission-blocking drugs, by taking advantage of the wealth of clinical data available for sildenafil, which been approved by the Food and Drugs Administration and is widely used in humans with little side effects to treat erectile dysfunction. In opposition to the Ehrlich’s “magic bullet” which consists of targeting pathways that are essential for parasites, but absent in humans, this strategy, referred to as “inverted silver bullet” [52], open new avenues towards the design of novel interventions to halt the spread of malaria to humans.

## Materials and Methods

### Gametocyte culture and stage specific purification

The *P*. *falciparum* clonal lines B10 and 3D7 (clones of NF54), and the transgenic lines *pHL-pfpka-r*, and *PfPDEδ- clone 4* have been described elsewhere [25,42,53]. Parasites were cultivated *in vitro* under standard conditions using RPMI 1640 medium supplemented with 10% heat-inactivated human serum and human erythrocytes at a 5% haematocrit [54]. Synchronous production of highly specific gametocytes stages was achieved according to described protocol [55]. For the isolation of gametocytes, culture medium was supplemented with 50mM (final concentration) *N*-acetylglucosamine (GlcNAc) from day 0 onwards and medium replacement was continued for 5 days to eliminate the asexual stages. Gametocyte preparations were enriched in different experiments by magnetic isolation using a MACS depletion column (Miltenyi Biotec) in conjunction with a magnetic separator.

### Measurement of intracellular cAMP levels and cGMP levels

To measure cAMP and cGMP levels, 6.10^6^ GIE were purified by magnetic isolation and the cell pellets were incubated with drugs at 37°C, centrifuged at 1,500 x g for 5 min and washed with PBS. Sample diluent containing detergents to lyse the cells, inactivate endogenous phosphodiesterases and stabilize the cyclic nucleotides was added to the pellet for 10 min at room temperature, as described in the kits protocol (FluoProbes, powered by Interchim). To avoid eventual interference with the assay stemming from haemoglobin contained in the red blood cell lysate, proteins were precipitated with 5% trichloroacetic acid (TCA) for 10 min on ice, and the precipitate was removed by centrifugation at 1,500 x g for 10 min. The supernatant was carefully removed and transferred to a clean tube. TCA was removed by four successive extractions with water-saturated Diethyl ether. The cyclic nucleotides content was measured after acetylation using a commercially available cAMP High Sensitivity Chemiluminescent Assay Kit or cGMP High Sensitivity Chemiluminescent Assay Kit (FluoProbes, powered by Interchim), cyclic nucleotides content was expressed as picomoles of cAMP or cGMP per 4.10^7^ cells.

### Drug treatments

Synchronized cultures containing 1 to 5% GIE were incubated 15 min to 2 h at 37°C with 10 nM to 150 μM 8Br-cAMP (8-Bromide-cyclic adenosine-monophosphate), 100 to 150 μM forskolin, 65 μM zaprinast, 50 nM Calyculin A, 10 nM to 100 μM sildenafil citrate, 10 μM compound 2 (4-[7-[(dimethylamino)methyl]-2-(4-fluorphenyl)imidazo[1,2-*a*]pyridin-3-yl]pyrimidin-2-amine), 10 μM H89, 10 μM KT5720, 10 μM PKI-m (Protein Kinase Inhibitor myristoylated), 10 μM GGTI (GeranylGeranylTransferase I) 298 trifluoroacetate salt hydrate. None of the compounds reported above, except Compound 2, PKI-m and KT5720, affected stage V GIE viability measured as parasite lactate dehydrogenase (pLDH) levels [56]. Stage V GIE were treated with compounds at the highest dose, for the indicated times, washed to remove the compounds and assayed for pLDH activity both immediately and after 72 h incubation at 37°C.

All reagents were purchased from Sigma-Aldrich or Euromedex, except Compound 2 that was provided by DB.

### Microsphiltration

Calibrated metal microspheres (96.50% tin, 3.00% silver, and 0.50% copper; Industrie des Poudres Sphériques) with 2 different size distributions (5- to 15-μm-diameter and 15- to 25-μm-diameter) composed a matrix used to assay infected erythrocyte deformability under flow, as described [28,29]. Suspensions of synchronized cultures containing 1% to 5% GIEs were perfused through the microsphere matrix at a flow rate of 60 mL/h using an electric pump (Syramed _sp6000, Arcomed_ Ag), followed by a wash with 5 mL of complete medium. The upstream and downstream samples were collected and smeared onto glass slides for staining with Giemsa reagent, and parasitaemia was assayed by counting 2000 erythrocytes to determine parasite retention versus flow-through. Retention rates of uninfected erythrocytes were monitored after labelling a subpopulation of erythrocytes with PKH67 (Sigma-Aldrich) according to manufacturer’s instructions. The proportion of labeled erythrocytes in upstream and downstream samples was determined by fluorescence microscopy using a Leica DM 5000 B at 100X magnification.

### Determination of GIE shape during microsphiltration

To visualize GIE shape during their flowing through the matrix, 1 mL of PBS/4% paraformaldehyde was added after perfusion of the GIE-containing culture on the microsphere matrix. After 5 min of incubation, fixed GIEs were separated from the microspheres by a 3-step decantation procedure, and GIE morphology was observed on a glass slide by light microscopy using a Leica DM 5000 B at 100X magnification. Microsphiltration experiments were performed in triplicate and 100 cells were counted per experiment.

### PDE assays

PDE activity in native parasite fractions was measured using a modification of a previously published method [57]. Briefly, parasites were frozen in liquid nitrogen and stored at -80°C until use. Parasites were resuspended in 500 μl lysis buffer (20 mM hepes and 250 mM sucrose, pH 7.0), subjected to 5 cycles of freeze-thaw in liquid nitrogen and pelleted at 100,000 *g* for 30 min. Particulate fractions were resuspended in lysis buffer containing EDTA-free protease inhibitors (Roche). PDE assays were carried out in triplicate wells of a 96-well plate in the presence of [^3^H]-labelled cGMP or cAMP (GE Healthcare) for 30 min at 37°C. Reactions were terminated by boiling the plate for 1 min, followed by a 3 min centrifugation at 900 *g*. 1 unit of alkaline phosphatase was added to each well and incubated for 30 min at 37°C. [^3^H]-labelled guanosine was separated from the radiolabelled cAMP/cGMP substrate using ion exchange (BioRad AG 1 x 8 resin). Supernatants containing the [^3^H]-labelled guanosine product were added to scintillation fluid (Optiphase Supermix, Wallac). Scintillation was measured using a Wallac 1450 Microbeta Liquid Scintillation Counter (Perkin Elmer) and PDE activity was expressed in pmol cAMP or cGMP/min/mg protein. Inhibition assays were carried out in the presence of compounds dissolved in DMSO. PDE assays for specific activity and IC_50_ determination were carried out at a native lysate dilution that gave 30% cGMP/cAMP hydrolysis.

### RNA isolation and transcript expression analysis by real-time RT-PCR

RNA was isolated from purified GIE or asexual stages using Trizol (Invitrogen) according to the manufacturer’s instructions and treated with DNAse I (Roche). RNA was reverse-transcribed using Superscript II that was primed with random hexanucleotides (Invitrogen). Real-time PCR was performed using an ABI Prism 7900HT sequence detector (Applied Biosystems). Relative quantification of cDNA was performed using 2ΔCt method (User Bulletin 2, ABI, http://www.appliedbiosystems.com). Triplicate PCR reactions were analyzed for each sample. Transmission-blocking antigen precursor *Pfs48/45* (*PF13_0247)* and ookinete surface antigen precursor *Pfs25* (*PF10_0303)* were used as markers of stage III and stage V gametocytes, respectively. Transcript abundance was compared using mean of ΔCt values calculated using ubiquitin-conjugating enzyme *(PF08_0085*) transcript (*HK* gene) as endogenous normalizer. Gene-specific primers used to profile the expression of *PDEα*, *PDEβ*, *PDEγ* and *PDEδ* were published in Wentzinger et al (2008) [39], and gene-specific primers used to profile the expression of *Pfs48/45*, *Pfs25 and HK* were published in Joice et al (2013) [6].

### Western-blotting analyses

GIE were purified by magnetic isolation and pelleted by centrifugation at 1800rpm. To prepare membrane extracts, 1.10^7^ GIE were resuspended in 100 μl of PBS1X/1%Triton X-100, freezed at -80°C overnight and centrifugated at 16000 *g* for 5 min at 4°C. To prepare total extracts, 5.10^6^ GIE were used. Pellets were denatured in protein loading buffer 5 min at 100°C and were separated by 4–12% SDS-PAGE, transferred to PVDF membrane and blocked for 1 h in 5% nonfat dry milk. Immunoblots were probed overnight with a purified rabbit antiserum against *Pf*PKA-R at 1:16, a rabbit mAb anti-phospho-PKA substrate (RRXS*/T*, 100G7E, Cell Signaling) at 1/1000, a mouse mAb anti-HSP70 antibody at 1/1000, or a mouse mAb anti-Band3 antibody (Sigma) at 1/5000 followed by 1 hour with horseradish peroxidase-conjugated anti-mouse or anti-rabbit IgG secondary antibodies (Promega) at 1:10 000. Detection step was performed using the Pierce chemoluminescence system (Pierce) following the manufacturer’s instructions. The levels of *Pf*PKA-R or phospho-PKA were quantified by densitometry using the Quantity One analysis software (BioRad). For each sample, we then calculated the ratio of protein levels relative to loading control HSP70 or Band 3.

### Indirect immunofluorescence microscopy

GIE were air-dried on glass blood smears and methanol-fixed for 10 min at -20°C. After 1h pre-incubation in PBS1X/2% BSA, slides were incubated overnight with a purified rabbit antiserum against *Pf*PKA-R at 1/50 and with AlexaFluor 594-conjugated goat anti-rabbit affinity-purified IgG (Molecular Probes) for 1 hour. Parasite nuclei were stained with Hoechst 33342 (diluted 1:20000, Life technologies). Samples were observed at 100X magnification using a Leica DM 5000 B.

### Statistical analysis

Statistical significance for differences in cAMP concentration and in protein levels was established using student test and Wilcoxon Mann-Whitney rank sum test. Statistical significance for differences in retention rates was established using Wilcoxon Mann-Whitney rank sum test. Statistical significance for differences in proportion of GIE showing different shape was established using a Chi-square test.

## Supporting Information

S1 Fig Stage V GIE deformability is not altered by H89. Retention in microsphilters of stages V GIEs. GIEs were pre-incubated at 37°C 30 min with 10 μM H89 or 0,1% DMSO (Control). Outliers are shown as open circles. ns: non-significant differences in retention rates compared to control. n: number of experiments.
(TIF)

S2 Fig GIE filterability is dependent on cGMP concentration. **A.** Stage V GIE were harvested by magnetic isolation and incubated at 37°C 30 min with 100 μM sildenafil, or 0.1% DMSO (Control). The total intracellular cGMP concentration was measured on aliquots of 6.10^6^ cells in duplicate wells. The assay was carried out two times. Error bars denote the standard error of the mean. **B.** Retention rates in microsphilters of stage V GIE (light grey) and uninfected red blood cells (uRBC, dark grey) pre-incubated 15 min at 37°C with different concentrations of 8Br-cGMP. The assay was carried out at least three times at each 8Br-cGMP concentration. Error bars denote the standard error of the mean. Outliers are shown as open circles.
(TIF)
