# Supplementary figures and images for: cAMP-Signalling Regulates Gametocyte-Infected Erythrocyte Deformability Required for Malaria Parasite Transmission
Source: PLoS Pathog. 2015 May 7;11(5):e1004815. doi: 10.1371/journal.ppat.1004815 (PMC4423841; doi:10.1371/journal.ppat.1004815)

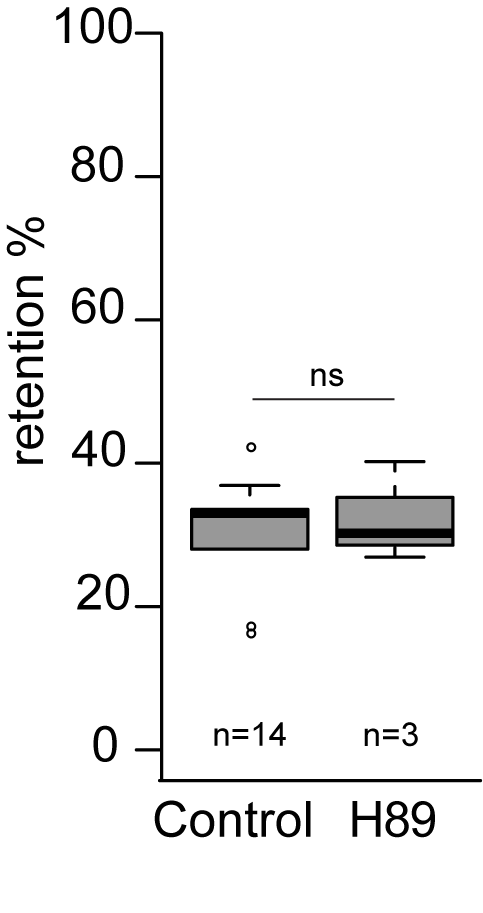

Supplement: S1 Fig — Retention in microsphilters of stages V GIEs. GIEs were pre-incubated at 37°C 30 min with 10 μM H89 or 0,1% DMSO (Control). Outliers are shown as open circles. ns: non-significant differences in retention rates compared to control. n: number of experiments. (TIF) [file ppat.1004815.s001.tif]

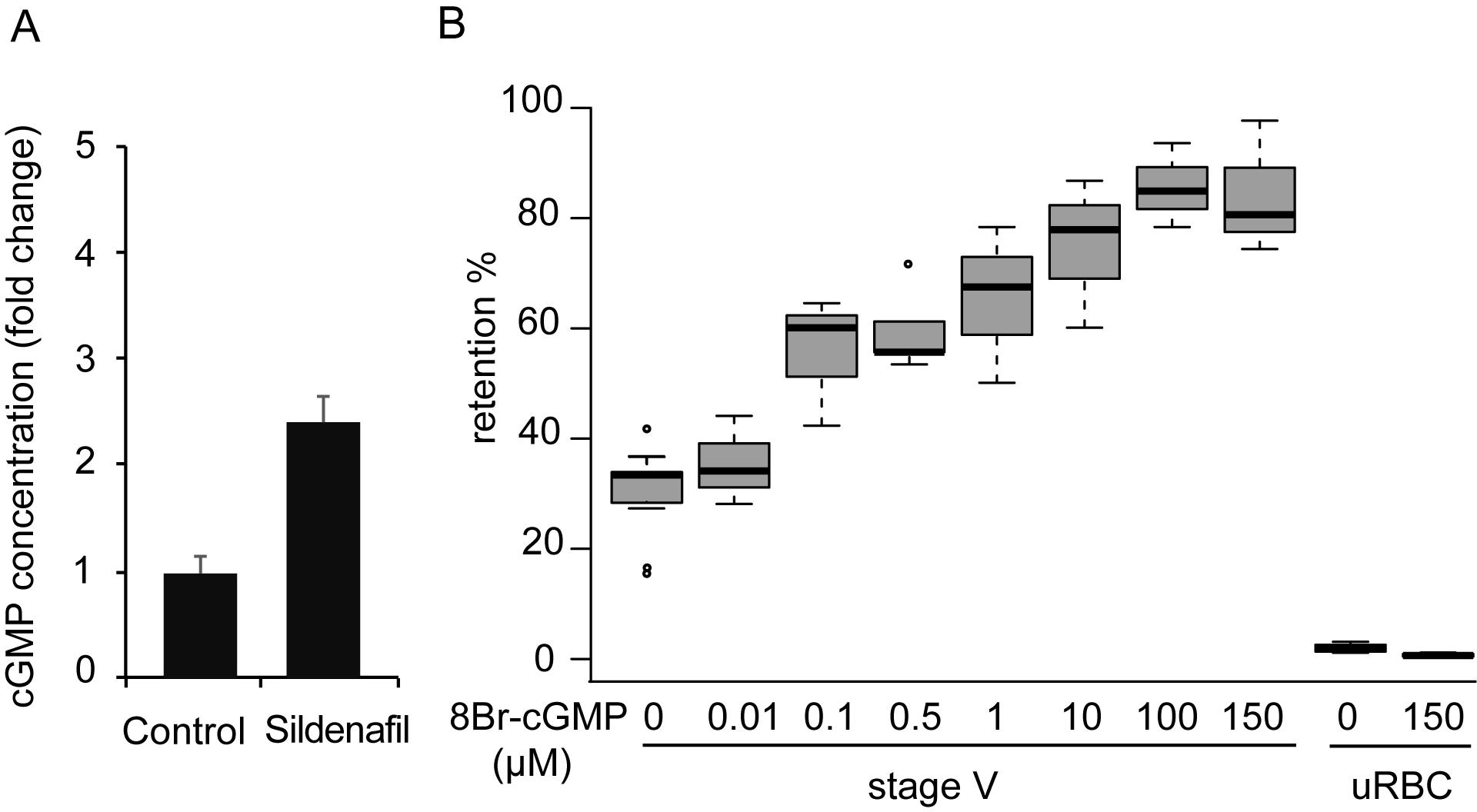

Supplement: S2 Fig — A. Stage V GIE were harvested by magnetic isolation and incubated at 37°C 30 min with 100 μM sildenafil, or 0.1% DMSO (Control). The total intracellular cGMP concentration was measured on aliquots of 6.106 cells in duplicate wells. The assay was carried out two times. Error bars denote the standard error of the mean. B. Retention rates in microsphilters of stage V GIE (light grey) and uninfected red blood cells (uRBC, dark grey) pre-incubated 15 min at 37°C with different concentrations of 8Br-cGMP. The assay was carried out at least three times at each 8Br-cGMP concentration. Error bars denote the standard error of the mean. Outliers are shown as open circles. (TIF) [file ppat.1004815.s002.tif]
